# An Intelligent Bio-Inspired Autonomous Surveillance System Using Underwater Sensor Networks

**DOI:** 10.3390/s23187839

**Published:** 2023-09-12

**Authors:** Shadab Khan, Yash Veer Singh, Prasant Singh Yadav, Vishnu Sharma, Chia-Chen Lin, Ki-Hyun Jung

**Affiliations:** 1 Department of Computer Science & Engineering, ABES Engineering College, Ghaziabad 201009, India; shadab1212@gmail.com; 2 Department of Computer Science & Engineering, Galgotias College of Engineering and Technology, Greater Noida 201310, India; yashveersingh85@gmail.com (Y.V.S.); vishnu.sharma@galgotiacollege.edu (V.S.); 3 Department of Computer Science and Engineering, Mahamaya Polytechnic of Information Technology (Govt.), Hathras 204102, India; pyaduvanshimitusa@gmail.com; 4 Department of Computer Science and Information Engineering, National Chin-Yi University, No.57, Sec. 2, Zhongshan Rd., Taiping Dist., Taichung 411030, Taiwan; 5 Department of Software Convergence, Andong National University, Andong 36729, Republic of Korea; kingjung@anu.ac.kr

**Keywords:** bio-inspired algorithms, cluster heads, energy efficiency, routing, underwater sensor networks

## Abstract

Energy efficiency is important for underwater sensor networks. Designing such networks is challenging due to underwater environmental traits that hinder network lifespan extension. Unlike terrestrial protocols, underwater settings require novel protocols due to slower signal propagation. To enhance energy efficiency in underwater sensor networks, ongoing research concentrates on developing innovative solutions. Thus, in this paper, an intelligent bio-inspired autonomous surveillance system using underwater sensor networks is proposed as an efficient method for data communication. The tunicate swarm algorithm is used for the election of the cluster heads by considering different parameters such as energy, distance, and density. Each layer has several clusters, each of which is led by a cluster head that continuously rotates in response to the fitness values of the SNs using the tunicate swarm algorithm. The performance of the proposed protocol is compared with existing methods such as EE-LHCR, EE-DBR, and DBR, and results show the network’s lifespan is improved by the proposed work. Due to the effective fitness parameters during cluster head elections, our suggested protocol may more effectively achieve energy balance, resulting in a longer network lifespan.

## 1. Introduction

In the arrangement of seas, rivers, oceans, and canals, water accounts for about 75% of the earth’s surface area. There are likely several undiscovered, valuable resources buried under the ocean’s surface. Exploratory endeavors have traditionally relied heavily on technology for their success. Previously, it was difficult to conduct underwater excursions utilizing sensors at all depths; however, recent technological breakthroughs have made it feasible. As a result, underwater sensor networks are being used as a kind of technology to facilitate underwater research. The underwater wireless sensor network (UWSN) combines wireless technology with a tremendously tiny micromechanical sensor to conduct intelligent sensing, intelligent processing, and intelligent communication [1]. The UWSN is a geographically dispersed network of autonomous SNs that monitors water-related variables such as temperature, pressure, and quality. The uncovered data may be used for a variety of applications that can be used to the advantage of individuals. Whether mobile or stationary, SNs are wirelessly linked to one another through communication modules [2] to convey a range of interesting events. The majority of underwater communication is handled through a network of nodes that communicate data to buoyant gateway nodes, which subsequently transport the data to the most convenient remote station [3]. At UWSN, acoustic transceivers are used for communication the majority of the time. Despite their narrow bandwidth and low frequency, acoustic waves possess quite long wavelengths. Therefore, information may be sent over kilometers using sonic waves, which have a vast range.

UWSNs are used for a variety of purposes, including the preservation of coasts, the monitoring of underwater pollution, the promotion of water-based activities, and the prevention of water-related catastrophes. Other responsibilities include monitoring the maritime environment for scientific research and economic utilization. UWSN is a prospective solution that can meet the requirements of ever-demanding applications. On the other hand, working with UWSN applications may be both enjoyable and difficult. This is because it is impossible to forecast the characteristics of the aquatic environment, which makes the design and implementation of such networks very complex.

UWSNs, also known as underwater sensor networks, are comprised of a variable number of nodes and various pieces of equipment that are dispersed over an underwater region of interest. The so-called deployed nodes and vehicles collaborate to perform various monitoring activities in this area [4]. SNs that are submerged in bodies of water enable several applications to function well. The collection of data from water and the sea, the extraction of oil from distant places, early warning systems to prevent the development of natural disasters, navigational assistance, and military surveillance are a few of these applications. Variation distinguishes the aquatic environment [4,5,6,7,8]. Changes in chemical composition, current strength, temperature, pressure, and a range of other variables are possible continuously. Self-coordination is essential for a node to function individualistically with existing SNs by exchanging data on several elements, such as depth, the spatial procedure of nodes that may vary with water currents, and packet forwarding with the base station.

This may be accomplished by using a node’s capabilities for self-communication. To effectively monitor and communicate in a dynamic environment such as this, the SNs comprising an underwater network must be adaptable, self-organizing, and autonomous. As radio signals, the transmission medium of typical ground-based sensor networks, are quickly absorbed by water, underwater wireless sensor networks (UWSNs) rely largely on auditory communication channels. Due to the circumstances underwater, acoustic modems are subjected to greater stress, which increases the amount of energy required for data transmission. Consequently, it is challenging and essential to discover methods for preserving power efficiency in the design of UWSNs. [9,10]. Low frequency, a longer wavelength, and a narrower bandwidth are typical characteristics of the acoustic waves created by UWSNs. Due to their characteristics, acoustic channels are favored for long-distance communication. The UWSN node that is geographically closest to an underwater event is the one that gathers data and transmits it across an auditory channel to the node that is closest to it. This process is continued until the information conveyed over a network of nodes reaches the surface-based sinks. To survive, a network of underwater sensors must adapt and reorganize due to the increasing forces present in the underwater environment [11,12,13]. Self-optimization is crucial for UWSNs to decrease power drainage and, as a result, increase the network’s lifetime [14,15]. Among other things, this is because of problems with power consumption, propagation delays, and synchronization. This would allow UWSNs to provide services in time-sensitive application situations. This paper contributes to the existing body of information on UWSNs by proposing a strategy for UWSNs that is both energy-efficient and extends the network’s lifetime. The proposed protocol is designed to maintain symmetrical energy drainage across all nodes in a cluster, thereby extending the lifetime of the network using the tunicate swarm algorithm (TSA) [16]. This work adds to the existing body of knowledge on routing protocol difficulties associated with energy efficiency in UWSNs. This was accomplished by combining the layering of nodes and the rotation of cluster heads using different fitness parameters. The major contributions of this paper are summarized as follows:

- To propose an intelligent bio-inspired Tunicate Swarm Algorithm (TSA) based autonomous surveillance system using underwater sensor networks as an efficient method for data communication.
- TSA is utilized to identify the optimal number of cluster heads based on the novel fitness function to enhance the network’s lifetime.
- The fitness function takes into account critical parameters such as inter-cluster distance, residual energy of nodes, and node density, aiding in the identification of the most suitable cluster head.
- To show the effectiveness of the proposed approach, the simulation results are compared with state-of-the-art techniques.

The remaining sections of the essay are structured as follows: In Section 2, the overall structure of UWSNs is dissected and investigated. In Section 3, the system model for UWSN is presented. The proposed methodology is given in Section 4. In Section 5, we go into further depth regarding the opportunities and obstacles in terms of simulation results that UWSN faces. Section 6 summary and conclusions bring the paper to a close.

## 2. Related Works

Determining a network’s routing configuration is one of the most important setup processes, and UWSN researchers have been hard at work developing various cutting-edge routing protocols over the last few years. These protocols have introduced novel techniques and concepts to a wide variety of subdomains that are now garnering considerable interest in networking literature. The vector used by vector-based forwarding (VBF) is crucial for easing the flow of information from the initial node to the sink node, which is upwards in the direction of the sink floating on the surface of the water. The use of this specific protocol results in a significant reduction in network traffic. Get on and off the bus [17]. Vector-based forwarding, also known as hop-by-hop (HH)-VBF, offers a change to the VBF protocol. As a contrast to the previously stated virtual buffer function, the virtual pipe or vector is defined before each hop. By allowing the intermediate node to pick the vector’s direction, the HH-VBF protocol ensures that forwarding may be delayed until a delivery route is established, even if there is only one accessible node within the communication range. To perform this, the intermediate node decides in which direction the vector will point. While transmitting data from one SN to another, the approach known as “depth-based routing” (DBR) [18] considers the relative depths of the SNs. Since a SN in UWSN delivers data packets downward to the sink node, a SN in DBR will not send a data packet to another SN until that node is at a shallower depth, indicating that it is closer to the surface so that a SN in a UWSN network’s DBR may relay data packets to the sink node. When a particular SN is often asked to provide data upstream because it is the node closest to the event of interest, DBR encounters a variety of issues, such as vacuum zones that hinder transmissions or unbalanced energy use. This might result in an imbalance in the node’s energy consumption. Using the previously described DBR protocol, data packets are sent via a feeding mode of transmission. This kind of transmission might result in channel occupancy and redundant data; however, the DBMR [19] protocol, which distributes data packets via a multi-hop protocol, eliminates these issues. Q-learning-based energy-efficient and lifetime-aware routing protocol (QELAR) [20] uses Q-learning, a member of the family of machine learning algorithms, to [21] extend the network’s lifetime. Q-learning seeks to reduce the load asymmetry by taking the remaining energy into account and reducing the total number of transmissions across the network. The PER [22] article employs both a node selector and a fuzzy logic framework. This critical selection applies the forward tree pruning approach to choosing which SN will transmit the data packets. In the E-PULRP [23,24] system, the nodes are placed in spherical shells that are concentric to one another. In the center of these concentric shells is a sink node, and each shell has a different energy level than the others. In the communication phase, the distance between each prospective relay node and the source is considered. The energy-efficient depth-based routing (EEDBR) [25,26] is a variant of the DBR that maintains high packing-to-delivery ratios. To perform this, the forwarding zones without a position advantage are shrunk in size. H2-DAB is a technique that is both lightweight and energy-efficient [27,28]. This protocol solves the problem of mobility, which is crucial given that the nodes under the water may move owing to currents. Each SN has a unique node ID and hop ID. The Hop ID represents the number of hops a data packet must traverse from the SN to the sink node. Since it is straightforward to obtain both the node ID and the hop ID, it is possible to dynamically update addresses even if nodes are moving. The rate at which this protocol’s addresses are updated is a characteristic that causes some concern. The VAPR [29] protocol analyses the number of node hops and the depth information when selecting where to proceed next. Even though UWSN makes it easy to obtain depth data, this method may make it difficult to obtain exact location data. During the improved beaconing phase, the beacon information, which may include the hop count, depth details, and the direction in which data are sent, is imprinted into each SN. During the second phase, SNs transmit data packets in the direction in which the data will be carried on the next hop. The construction of an ideal depth function that facilitates frequent depth threshold alterations is one manner through which AMCTD [30] extends the lifespan of the network. This is accomplished by discovering solutions to challenges such as load distribution and excessive energy use. Vacant nodes are pushed deeper in GEDAR [31,32] using depth adjustment techniques, and data packets are routed using a greedy opportunistic strategy. In the DRP [33] protocol, the transmission collision probability is taken into account while selecting the optimal route. In addition to a high transmission rate, the DRP-selected route has high residual energy. The lifetime of a network is computed by factoring in the percentage of its nodes that are active at any given moment and using that figure as input for the calculation. This fraction implicitly incorporates information about the remaining energy of network nodes when picking candidates that might serve as forwarding nodes. By considering the dependability of the connections, the network aims to extend its lifespan. The 2013 invention of improved energy-balanced routing (IE-BR) solves the issue of insufficient global energy use in UWSNs. The first stage of the two-step protocol is establishing the routing, while the second step is transmitting the data. Throughout the routing process, underwater links between sensors are constructed using a mathematical model that selects neighbors that are at optimal distances and depths. Once the routing configuration is complete, the second process, which involves data transfer while maintaining an energy balance inside the network, begins. This is carried out by dynamically rotating the links formed in the first step based on the differences in energy levels (EL) between close nodes. In the cluster-based routing protocol known as EE-MDC-HSRP [34], the mobility factor, node density, and residual energy are employed to select the cluster heads. In EVAGR [35], an empty area is managed by a weight function to pick a set of forwarding nodes, and data transmission occurs in the chosen set of nodes. The weight function takes into consideration aspects such as energy usage and the number of candidate nodes. The potential nodes are chosen based on the packet transmission directed towards the sonobuoys. Datta et al., discussed a cluster head rotation-based routing protocol for UWSNs [21]. However, this method does not efficiently utilize the energy.

## 3. System Model

In the proposed underwater sensor networks, the varying numbers of nodes deployed in the underwater sensor network are classified using a three-level heterogeneity model. The nodes are categorized according to their remaining energy. The model proposed in this study is based on the following assumptions:

- The network consists of two types of nodes: underwater sensor (UWS) nodes, which are stationary and categorized into cluster heads and cluster member’s nodes due to the clustering approach, and a Sink Node located at the periphery of the surveillance area.
- The heterogeneous network is considered in the form of heterogeneous nodes’ energy.
- A single Sink Node is present within the network, and there are sufficient energy is available.
- The position of the Sink Node is assumed to be known and fixed throughout the surveillance period.
- Underwater sensor nodes possess limited energy and do not have their own energy supply.
- Regular underwater nodes share identical baseline energy levels and unique identification numbers.
- Node positions will be determined using a localization method.
- The communication range between UWS nodes is assumed to be fixed, and nodes can only communicate with each other if they are within this range.
- The network topology is assumed to be relatively static during the surveillance period, with minimal node mobility or changes in connectivity.
- Interference from external sources or neighboring networks is assumed to be negligible, allowing for efficient communication within the network.

These assumptions collectively form the basis for the modeling and simulation of the proposed intelligent bio-inspired Tunicate Swarm Algorithm (TSA) based autonomous surveillance system using underwater sensor networks. There are a total of n nodes, and three distinct forms of energy are present: form-1 ($f_{1}$), form-2 ($f_{2}$), and form-3 ($f_{3}$). The work has n nodes in total. The nodes with energy values of form-1 are low-energy SNs, while the nodes with energy values of form-3 are high-energy nodes. Due to their energy forms falling between those of form-1 and form-2, nodes with energy levels of form-2 satisfy the inequality form-1 < form-2 < form-3. The networks include more instances of nodes with low energy forms ($f_{1}$) than nodes with intermediate energy forms ($f_{2}$) and nodes with high energy forms ($f_{3}$). In contrast, the inequality form-3 < form-2 < form-1 is met since nodes with the highest energy have the fewest occurrences. The whole energy usage of the network [12] is considered as

(1) $${Sum}_{f}=î\times no_{of_{nodes}}\times f_{1}+{î}^{2}\times no_{of_{nodes}}\times f_{2}+(1-î-{î}^{2})\times no_{of_{nodes}}\times f_{3}$$

where î is represented as the model parameter and number of form-1, form-2 and form-3 nodes are $î\times no_{of_{nodes}}$, ${î}^{2}\times no_{of_{nodes}}$ and $\left(n_{n}-(î\times no_{of_{nodes}}+{î}^{2}\times no_{of_{nodes}})\right)$ with the $f_{1}$, $f_{2}$ and $f_{3}$ energy, respectively. 

By using î=0 in (1), only one category of nodes is generated with defined energy ${{Sum}_{f}=no_{of_{nodes}}\times f}_{3}$ in 1-level heterogeneity. These nodes have the highest energy in the networks with the minimum number of nodes, and that can be modified as follows:

(2) $$î=\frac{f_{3}-f_{1}}{\beta \times fun(f_{2},f_{3})}$$

Equation (1), gives two solutions by the $1-î-{î}^{2}=0$, i.e., $(\sqrt{5}-1)/2$ and $(\sqrt{5}+1)/2$. The expression $(\sqrt{5}-1)/2$ gives values between (0, 1) and define 2-levels of heterogeneity.

The upper bond is indicated as ${î}_{upper\_b}\;with\;value\;(\sqrt{5}-1)/2$ and consider the lower bound as ${î}_{lower\_b}$ where î satisfy the inequality ${î}_{lower\_b}<î<{î}_{upper\_b}$ in the case of 3-level heterogeneity and consider the function value fun as ${(f}_{3}-f_{2})$. Thus, calculate ${î}_{lower\_b}$ as below:

(3) $${î}_{lower\_b}<\frac{f_{3}-f_{1}}{\beta \times ({f_{3}-f}_{2})}<(\sqrt{5}-1)/2$$

Using relation ${î}_{lower\_b}<\frac{{\alpha_{2}+\alpha }_{1}}{\beta *\alpha_{2}}$ and consider $f_{2}=\alpha_{1}+f_{1}$ and $f_{3}=\alpha_{2}+f_{2}$ gives the below

(4) $$\frac{\alpha_{2}}{\alpha_{1}}<\frac{1}{\beta \times {î}_{lower\_b}-1}=-\frac{\alpha_{2}}{\alpha_{1}}\geq \frac{1}{1-\beta \times {î}_{lower\_b}}$$

If $-\frac{\alpha_{2}}{\alpha_{1}}=0$ in (4) we obtained the below relation:

(5) $$1-\beta \times {î}_{lower\_b}<0=\frac{1}{\beta }<{î}_{lower\_b}$$

By putting the value in (3) which gives as below:

(6) $${(f}_{3}-f_{1})\leq \frac{\beta \times \left(\sqrt{5}-1\right)}{2}\times (f_{3}{-f}_{2})$$

(7) $${\beta \times \left(\sqrt{5}-1\right){\times f}_{2}-{2\times f}_{1}\leq \left(\beta \times \left(\sqrt{5}-1\right)-2\right)\times f}_{3}$$

However, the energies of three types of nodes are fixed as $f_{1}$, $f_{2}=f_{1}\times (1+\omega )$ and $f_{3}=f_{1}\times (1+µ)$, respectively, where constants are ω=0.06 and µ=0.11.

The energy consumption model is considered for the consumption of energy for the deployed underwater sensor networks, as discussed in [36]. Let the consumption of energy during the reception of data be denoted as ${pow}_{rcvd}$, delay in sending m bit data are represented by $T_{m}$. The total energy required for transmitting m bit data over d distance is given as follows:

(8) $$E_{T}\left(m,d\right)=T_{m}\times {pow}_{rcvd}\times a(f)$$

where a(f) is the absorption coefficient.

The total energy required for receiving m bits of data over d distances is given as follows:

(9) $$E_{R}\left(m,d\right)=m\times {pow}_{proc}$$

where ${pow}_{proc}$ indicates the energy consumption in one-bit processing of acoustic SNs. 

The total energy required for aggregating m bit data are given as follows:

(10) $$E_{agg}\left(m\right)=m\times {pow}_{integtate}$$

where ${pow}_{integrate}$ indicating the energy consumption in the one-bit aggregation of acoustic SNs. 

## 4. Proposed Methodology

The operating stages of the proposed method are divided into two phases, namely network setup and data transmission. These two unique stages of network operation are separated and investigated individually. We began by discussing the time required to construct the network. At this step of the method, the nodes are randomly organized, and the sink is positioned precisely in the center of the rectangular network. There are three different energy levels for nodes, which leads to the conclusion that the nodes themselves are heterogeneous. 

### 4.1. Duration of Network Installation

We began by discussing the time required to construct the network. At this step of the method, the nodes are randomly organized, and the sink is positioned precisely in the center of the rectangular network. There are three different energy levels for nodes, which leads to the conclusion that the nodes themselves are heterogeneous. Once the network is operational, the nodes will begin to link with one another, and the CH selection process will commence. Before selecting the CH, we calculate the fitness function’s value, which will be utilized by the tunicate swarm algorithm (TSA) to determine each node’s fitness. This allows the TSA algorithm to choose the nodes with the highest probability of becoming CH. To serve as CH, the physically fittest node is chosen; this decision is based on fitness, which is based on establishing the fitness criteria. There are different parameters that are used to define the fitness function from the clustering point of view.

#### 4.1.1. The Ability of a Node to Create Energy

The availability of energy in a node is the most significant factor in determining whether or not it will be selected as a CH. Therefore, a node’s energy availability is controlled by its initial fitness value, also known as ${FitP}_{1}$. This is essential because the energy of a node decreases with each round. The previously described Equation (11) is utilized to compute the available energy of the ith node. The desire for a node to be CH grows as its ${FitP}_{1}$ value increases.

(11) $${FitP}_{1}=\sum_{i=1}^{n}E_{current}$$

#### 4.1.2. Node Energy

Due to the fact that the nodes’ energy levels span from form 1 to form 3, respectively, the initial vitality is high. Therefore, whenever possible, it is preferable to have CH selection from high energy nodes such as form 3. This is conducted to maintain CH for the longest amount of time feasible. Using Equation (12), the energy level of the ith node may be calculated. The desire for a node to be CH grows as its ${FitP}_{2}$ value increases.

(12) $${FitP}_{2}=\sum_{i=1}^{n}E_{i}$$

#### 4.1.3. The Percentage of the Distance between Each Node and the Sink

Frequently, the quantity of energy required to interact with a distant node increases. Therefore, a CH should be chosen from among the nodes that are located closest to the component, also known as the sink, that is responsible for data collection. It is good to save energy. Thus, the distance between the candidate node and the sink is considered by the third fitness parameter, denoted as ${FitP}_{3}$. The calculation of the distance to the sink is shown by Equation (13).

(13) $${FitP}_{3}=1/\sum_{i=1}^{n}{Distance}_{SN-Sink}$$

#### 4.1.4. The Distance between Neighboring CHs

This aspect becomes crucial after the first round. In the initial configuration, this option had a value of 0. Once the first CH has been chosen, however, the selection of the next CH is based on the distance between the candidate node and the already-selected CH. It contributes to the maintenance of the inter-cluster distance, which in turn is responsible for the network’s energy balance. The letter ${FitP}_{4}$ represents the distance between the candidate node and the CH node, which is determined by the fourth fitness parameter. Equation (14) provides the calculation for the Euclidean distance between the node and the CH.

(14) $${FitP}_{4}=1/\sum_{i=1}^{n}{Distance}_{SN-CH}$$

#### 4.1.5. Node Density

Since CH collects data from each node in a cluster, the number of nodes around the candidate node must be as high as possible. As a consequence, the distance between the selected CH and the other cluster nodes will be decrease. ${FitP}_{5}$, the fifth fitness statistic, considers the effective average distance between each node and the other nodes. The procedure will proceed with the energy of the chosen CH intact. Note that, to be included as a required component of the fitness function, all of the aforementioned fitness characteristics have been harmonized over the ranges [0–1]. The following Equation (15) is used to compute a node’s average distance from the other nodes in the cluster.

(15) $${FitP}_{5}=1/(1/C_{n})\times (\sum_{i=1}^{C_{n}}\sum_{rest=1}^{C_{n}}{Distance}_{i-rest}(i)))$$

Currently, the primary objective is to obtain the greatest Fit in order to obtain the highest selection of CH. Equation (16) illustrates the integration of all variables associated with fitness.

(16) $$Fit={FitP}_{1}\times µ+{FitP}_{2}\times µ+{FitP}_{3}\times µ+{FitP}_{4}\times µ+{FitP}_{5}\times µ$$

(17) $${µ}_{1}+{µ}_{2}+{µ}_{3}+{µ}_{4}+{µ}_{5}\;=\;1$$

The Equation (17) represents the total of all weight variables that are considered while choosing CH. The weight provided to each of these weight components is determined by the specific purpose for which the underwater sensor network was designed. 

### 4.2. Tunicate Swarm Based Optimized Cluster Head Selection Phase

The Tunicate Swarm Algorithm (TSA) stands as an innovative bio-derived metaheuristic technique. The algorithm’s blueprint draws inspiration from the tunicate swarm’s actions of jet propulsion and intelligent collective foraging. Initial emphasis lies on preventing clashes among exploration entities in the propulsion process. Subsequently, movement aligns with the optimal neighboring direction, culminating in convergence towards the premier search agent. The following steps are used to find the optimized CHs in the proposed network.

#### 4.2.1. Evade Clashes Amid Exploration Entities (SAs) Vector

In order to ascertain the fresh position of the search agent and prevent clashes amidst SAs, Vector $\vec{A}$ is computed employing the subsequent Equation (18).

(18) $$\vec{AC}=\vec{GV}/\vec{SF}$$

where $\vec{GV}$ symbolizes the force of gravity, which can be calculated as presented by Kaur et al. [16], The computation of social forces $\vec{SF}$ among the SAs is carried out using the subsequent Equation (19).

(19) $$\vec{SF}=[{Min}_{s}+r_{3}\times ({Max}_{s}-{Min}_{s})]$$

where ${Min}_{s}$ and ${Max}_{s}$ denote the initial and subsequent velocities of social interaction, respectively.

#### 4.2.2. Proceed in the Direction of the Optimal Neighboring Search Agent

Subsequent to conflict avoidance, the ensuing equation is employed to mathematically articulate this phenomenon.

(20) $$\vec{Dst}=\left|{Pos}_{FS}-{rand}_{1}\times \vec{{Pos}_{T}}(x)\right|$$

$\vec{Dst}$ is the distance vector between the position of food source ${(P}_{FS})$ and position of tunicate SA $\left(\vec{P_{T}}\right)$. x is the current iteration, $r_{4}$ is a random number in the range from 0 to 1 and || is used for absolute value.

$\vec{Dst}$ stands for the distance vector spanning between the location of the food source (${Pos}_{FS}$) and the position of the tunicate search agent ($\vec{{Pos}_{T}}$). The variable x signifies the current iteration, ${rand}_{1}$ denotes a random value within the range of 0 to 1.

#### 4.2.3. Approach towards the Optimal Search Agent

To express this phenomenon mathematically, the following equation shows the optimal solution as the updated position ($\vec{P_{T}}\left(x\right))$ of tunicate SAs towards the food source.

To mathematically represent this phenomenon, the following equation demonstrates the optimal solution as the revised position $\vec{{Pos}_{T}}\left(x\right)$ of tunicate search agents moving towards the food source.

(21) $$\vec{{Pos}_{T}}\left(x\right)={Pos}_{FS}+\vec{A}\times \vec{D}\;if\;r_{4}\geq 0.5$$

(22) $$\vec{{Pos}_{T}}\left(x\right)={Pos}_{FS}-\vec{A}\times \vec{D}\;if\;r_{4}\leq 0.5$$

#### 4.2.4. Swarm Dynamics Behavior

The tunicate demonstrates collective behavior, a concept characterized by the synchronization of other search agents’ positions with those of the superior search agent. The mathematical expression of the tunicate’s collective behavior is depicted as follows:

(23) $$\vec{{Pos}_{T}}\left(x+1\right)=[\{\vec{{Pos}_{T}}\left(x\right)+\vec{{Pos}_{T}}\left(x+1\right)\}]/(2+r_{3})]$$

Algorithm 1 shows the Tunicate Swarm based optimized cluster head selection process as follows:
**Algorithm 1:** Tunicate Swarm-Based Optimized Cluster Head Selection AlgorithmInput: Initial quantity of individuals SNs in the population of
$T_{i}\forall i=\left(1,2,\ldots ,n\right)$Output: Optimal selction of CHs base on fitness of SA (${OFit}_{SA}$)  //Initiate the Tunicate Swarm Algorithm process1. Set the initial values of parameters $\vec{AC},\vec{GV},\vec{SF}$ and Max_I2. Set $\mathrm{Cur\_I}\leftarrow 0,{Min}_{s}\leftarrow 1\;and\;{Max}_{s}\leftarrow 4$3. **While **(Cur_I=Max_I)
** Do**4.    **For each**
k←1 to 2
**Do**5.       ${OFit}_{SA}$ ← Assess the fitness of every search agent by employing Equation (16)6.       Calculate $\vec{AC}$, $\vec{SF}$ and $\vec{Dst}$ using Equations (18)–(20) respectivelly7.      **if**
${rand}_{1}\geq 0.5$ then8.        The new location $\vec{{Pos}_{T}}\left(x\right)$ is calculated using Equation (21)9.      **else**10.        The new location $\vec{{Pos}_{T}}\left(x\right)$ is calculated using Equation (22)11.     **end if**12.     **end for**13.   The current location of SNs are updated using Equation (23)14.   Update the values of $\vec{AC},\vec{GV},and\;\vec{SF}$15.  Cur_II=Cur_I+116.  **end while**17. **return **${OFit}_{SA}$** as the CH****End TSA procedure**

### 4.3. The Data Transfer Phase

During this phase, data are transferred from the cluster nodes to the CH. The data are then sent from the CH to the sink, where it is finally transmitted online to the user. It should be emphasized that single-hop communication is utilized throughout the entire procedure. As a result, the network no longer has issues with hot spots.

The complete working process of the proposed intelligent bio-inspired autonomous surveillance system using underwater sensor networks is defined as follows in Algorithm 2:
**Algorithm 2:** Complete Working Process of the Proposed Method$\mathrm{Input}:\;no_{of_{nodes}}=100\;to\;800$, ${round}_{max}=maximum\;rounds$, monitoring area=1000 m×1000 m×1000 m$\mathrm{Output}:\;{node}_{alive}$$,\;{node}_{dead}$, ${node}_{CH}$, and throughput1.Sensors are placed in the underwater area i.e., 1000 m×1000 m×1000 m in the form of heterogeneous networks2.$\mathrm{Set}\;{node}_{CH}=0$$,\;E_{i}=0$, and $E_{residual}$3.for p $=1\;\mathrm{to}\;{round}_{max}$ do 4.   $\mathrm{Set}\;{node}_{alive=no_{of_{nodes}}}and$${\;node}_{dead}=0$5.for j=1 $\mathrm{to}\;no_{of_{nodes}}$ do6.  $\mathrm{if}\;E_{residual}\left(j\right)>0$ then7.  Execute the election procedure of cluster heads as defined in Algorithm 18.${node}_{CH}$$={node}_{CH}$ + 19.  Enforce a steady phase for transmission and collection of the sensed data from the underwater area10.else11.  Update the energy dissipation
$E_{residual}\left(j\right)$ of the node based on the utilization as defined by the energy model12.if
$E_{residual}(j)==0$ then13.  ${node}_{dead}$$={node}_{dead}$ + 114.if
${node}_{dead}==no_{of_{nodes}}$ then15.  all available SNs are dead16.  break17.Else18.  ${node}_{alive}={node}_{alive}-{node}_{dead}$19.end if20.end for21.end for22.return j 

The proposed methodology encompasses two distinct phases: network setup and data transmission. This study commences with the examination of the time required for network establishment, characterized by the random arrangement of nodes and the precise positioning of the sink at the network’s center. Within this configuration, nodes exhibit three varying energy levels, resulting in a heterogeneous node population.

In the network setup phase, the critical factors affecting cluster head selection are evaluated through a fitness function. This function includes parameters such as the node’s energy availability, energy levels, distance to the sink, distance between neighboring CHs, and node density. These factors collectively contribute to a fitness value that drives CH selection. Subsequently, the Tunicate Swarm Algorithm is introduced to optimize the cluster head selection process. TSA draws inspiration from the tunicate swarm’s behaviors and employs collective foraging and propulsion tactics to enhance the selection process.

Moving into the data transfer phase, data are relayed from cluster nodes to CHs and then to the sink for ultimate delivery to users. Notably, single-hop communication is employed throughout, mitigating issues related to hotspots and promoting efficient data transmission.

The complete working process of the proposed intelligent bio-inspired autonomous surveillance system utilizing underwater sensor networks is summarized through a comprehensive algorithm. This algorithm outlines the steps required for network deployment, cluster head selection, energy management, and data transfer.

In summary, the proposed methodology encompasses network setup, data transmission, and a sophisticated Tunicate Swarm Algorithm to optimize cluster head selection. Through a well-defined algorithm, this study establishes a systematic framework for the implementation and assessment of the intelligent bio-inspired autonomous surveillance system within underwater sensor networks.

## 5. Simulation Results

This section assesses the performance of the proposed protocol by comparing it to that of three prominent UWSN protocols: DBR [18], EE-DBR [25,26], and EE-LHCR [37]. The performance is evaluated assuming a three-dimensional environment with dimensions of 1000 m×1000 m×1000 m and a total of 100 nodes distributed over 200-m-high virtual layers. Aqua-Sim, a network simulator that is an NS2.30 adaptation of an underwater simulator, is used for all programming. The simulation parameters are given as transmission range, data packet size, initial energy, power consumption for transmission mode, and power consumption for receive mode: 250 m, 50 bytes, 10,000 μj, 2.0 μj, and 0.5 μj, respectively. The performance of the proposed protocol is evaluated using the four metrics listed below: It is referred to as the network’s energy consumption when a network of SNs transfers data packets from source to sink. The delivery ratio is derived by comparing the total number of packets transmitted by the source node to the total number of packets received by the sink node. End-to-end latency is the time a packet takes to travel from its source node to its destination node. The duration between the death of the first node in a network and the death of the last node in that network is referred to as the “network lifetime.” Theoretically, a network may continue to function even after the death of its last node. Table 1 shows the parameter values for the simulation.

### 5.1. Utilizing Energy That Is Present

**Figure 1:**
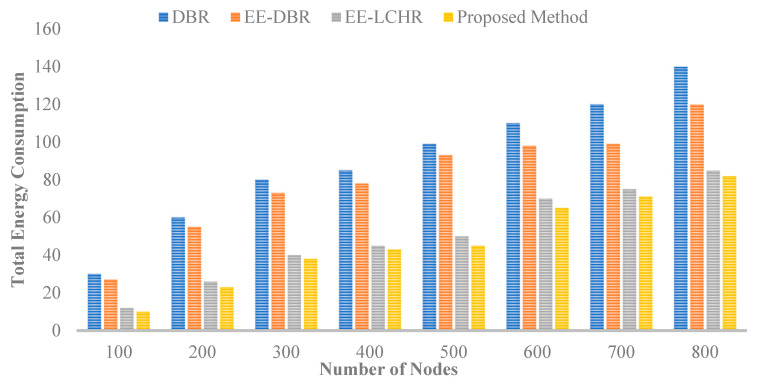
Total energy consumptions vs. number of nodes.

The overall quantity of energy used is the sum of the energies necessary for data packet transmission and reception, as well as the energies required to send out inquiry broadcasts, which are used to identify who will be the next to transmit data packets. Figure 1 compares the average energy consumption of the proposed protocol to that of the DBR [18], EE-DBR [25,26], and EE-LHCR [37], which may be found. The proposed protocol requires less energy to transport packets since it selects the forwarding relay set for each transmission based on the fitness value of the cluster heads. This value is determined after every communication. Compared to other protocols, the proposed protocol has a greater packet delivery ratio due to the dependence of the holding time on the Euclidean distance between cluster heads. This is the case since it is not used by various protocols.

### 5.2. The Proportion of Successfully Delivered Packages

**Figure 2:**
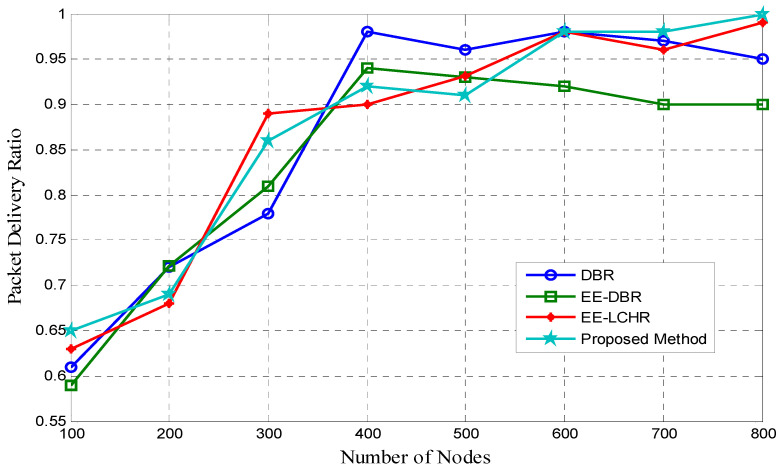
Packet delivery ratio vs. number of nodes.

Figure 2 illustrates the different packet delivery ratios for proposed and existing EE-LCHR [37], DBR [18], and EE-DBR [25,26] methods. The packet delivery ratio of the proposed method converges to that of DBR [18], EE-DBR [25,26], and EE-LCHR [37], exhibiting a minor improvement over these other ratios as a result of an increase in the number of available nodes caused by an increase in network lifespan. When there are fewer nodes in the network, the proposed method of routing has a greater packet delivery ratio compared to conventional routing protocols. The proposed method packet delivery rate is equivalent to the DBR [18], EE-DBR [25,26], and EE-LHCR [37] rates.

### 5.3. Beginning to End Delay

**Figure 3:**
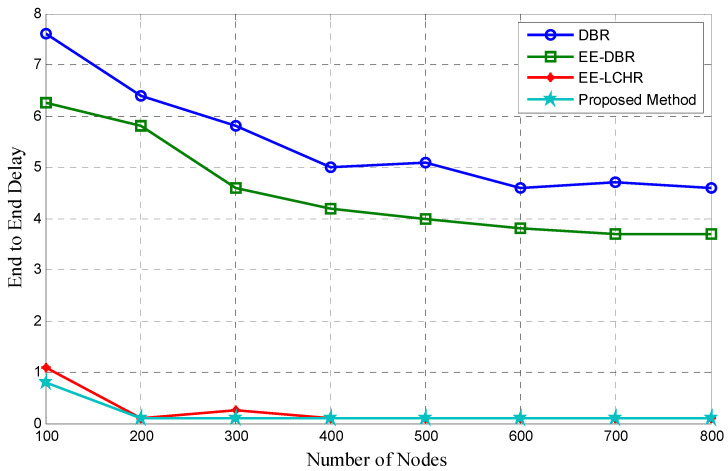
End-to-end delay vs. number of nodes.

Figure 3 demonstrates that the hat proposed method has the lowest end-to-end latency in comparison to DBR [18], EE-DBR [25,26], and EE-LHCR [37] in terms of the time between the production of a packet at the source node and its arrival at the sink node. Since the number of nodes in a network rises, the time it takes from beginning to end reduces, as more nodes mean more nodes that are qualified for packet transmission are available. The efficacy of the proposed method is superior due to the fact that it minimizes the incidence of collisions between source and sink nodes during packet transmission.

### 5.4. Duration of the Network’s Existence

Network lifespan is the time from the network’s formation until it becomes inoperable due to the loss of one or more nodes due to mechanical failure or depletion of energy. As indicated in the preceding Section 3, the proposed protocol defines the network lifetime as the sum of all probable maximum path lifetime values inside a network. The maximum PLV for a certain route is the minimum period necessary to elapse before a cluster failure along that route makes that path inoperable. The proposed method has a greater theoretical network lifetime value than DBR [18], EE-DBR [25,26], and EE-LHCR [37], according to the simulation findings. In selecting the cluster head that will be responsible for delivering the data packet upwards in the proposed method, the amount of remaining energy indicated by the fitness value is considered.

**Figure 4:**
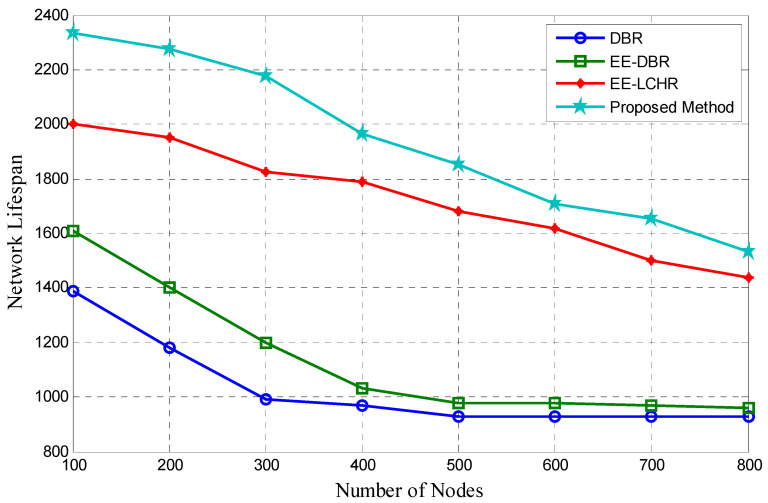
Network lifespan vs. number of nodes.

The rotation of the cluster head increases the lifetime of the network cluster because it avoids uneven energy distribution. When the lifespan of a cluster along a certain route is increased, the route in question is used for a longer amount of time after the augmentation. The network’s longevity, which is the total of the lifetimes of each of these other routes, shows a significant increase. As seen in Figure 4, the energy considerations included in the proposed protocol are a significant contributor to the network lifetime enhancement exhibited in the proposed method against DBR [18], EE-DBR [25,26], and EE-LCHR [37].

The proposed study introduces a novel energy-efficient routing protocol and compares its performance against existing methods. The proposed protocol demonstrates reduced energy consumption for data transmission thanks to the smart selection of relay sets based on cluster head fitness values. This approach yields a higher packet delivery ratio, outperforming other protocols due to its unique utilization of Euclidean distance. Moreover, the proposed protocol minimizes end-to-end delays through efficient collision management. It extends network lifespan by considering residual energy for cluster head selection, resulting in improved longevity compared to benchmark methods. In conclusion, the proposed protocol offers enhanced energy efficiency, packet delivery, latency, and network lifespan, highlighting its potential for advancing wireless network routing.

## 6. Conclusions

The proposed protocol is a routing-based technique developed for use with UWSNs in this work. The purpose of this effort was to achieve an ideal level of energy dissipation that extends the lifespan of the network. Using the proposed protocol, heterogeneous SNs are deployed in the monitoring area. Then, clusters are formed, and their heads rotate regularly depending on a function whose value is dictated by the quantity of residual energy in each node. During the last phase, data are sent depending on the proximity of a cluster head to a sink node within the communication range. Once the proposed protocol was implemented in Aqua-sim, an NS-2.3-based underwater simulator, simulations of the proposed protocol were conducted utilizing that simulator. The results of the 50 simulations indicate that the proposed protocol outperforms the DBR [18], EEDBR [25,26], and EE-LHCR [37] protocols on a variety of criteria. These findings were obtained using simulations. It has been shown that the proposed protocol outperforms DBR [18], EEDBR [25,26], and EE-LHCR [37] in terms of average end-to-end latency, packet delivery ratio, average network lifespan, and average energy consumption. The reason for this improvement is finding the different fitness parameters that reduce energy consumption. Future initiatives to lower the energy consumption of underwater sensor networks may involve the development of machine learning algorithms to recognize the cluster heads and the rotation of their nodes.

## Figures and Tables

**Table 1:** Simulation parameters.

Simulation Parameters	Values
Network area	1000 m×1000 m×1000 m
Number of nodes	100
Transmission range	250 m
data packet size	50 bytes
Node density/Energy heterogeneity (3 forms)	10% form-3 (2 j), 20% form-2 (1.5 j), 70% form-1 (1 j)
power consumption for transmission mode	2.0 μj
power consumption for receive mode	0.5 μj
Maximum Iteration	40

## Data Availability

No data has been used in this study. Moreover, all the information will be available on request to Yash Veer Singh.
